# Familial hemophagocytic phohistiocytosis induced by PRF1 mutation with neurologic manifestations as the initial clinical presentations: A case report

**DOI:** 10.1097/MD.0000000000034198

**Published:** 2023-06-30

**Authors:** Yang You, Wenjuan Wu, Baoguang Li

**Affiliations:** a Department of Imaging, The Fourth Hospital of Hebei Medical University, Shijiazhuang, China; b Department of Neurology, Hebei Children’s Hospital, Hebei Children’s Hospital Affiliated to Hebei Medical University, Shijiazhuang, China.

**Keywords:** case report, CNS neurological manifestations, familial hemophagocytic phohistiocytosis, leukoencephalopathy, perforin 1 gene

## Abstract

To investigate the clinical characteristics of familial hemophagocytic phohistiocytosis (FHL) induced by PRF1 gene mutation and with central nervous injury as the initial presentation.

**Case presentation:**

Herein, we presented 2 cases of a familial hemophagocytic syndrome caused by PRF1 gene mutation in 1 family with central nervous injury as the first symptom and searched relevant literature for clinical analysis of its pathogenic characteristics. Two children from 1 family were included in this study, both of whom had complex heterozygous mutations of C. 1189_1190dupTG (p.H398Afs*23) and C. 394G>A (p.G132R). Literature search further revealed 20 cases of PRF1 gene mutation-induced familial FHL with central nervous injury as the initial presentation. The main neurological symptoms included cranial nerve injury (81.8%), convulsion (77.3%), ataxia (63.6%), encephalopathy (59.1%), and limb paralysis (40.9%). Cranial imaging findings were dominated by the cerebral hemisphere (100%), cerebellar hemisphere (85%), brainstem (55%), and periventricular white matter (40%), and 73.7% of cases had elevated white blood cell count in CSF. Most cases were confirmed by differential diagnosis and gene sequencing, which suggested that C. 673C>T (P.r225W), C. 394G>A (P.G132r), C. 666C>A (p.H222Q), C. 1349C>T (p.T450M), C. 1349C>T (p.T450M), and C. 443C>C (p.A148G) could be focal mutations of this disease.

**Conclusion:**

Lesions involving the cerebellum and brainstem in children with ataxia and cranial nerve damage could be indicative of primary FHL; thus, the inherent immune test and gene test should be timely performed to help confirm the diagnosis, guide the treatment, and improve the prognosis.

## 1. Introduction

Primary hemophagocytic phohistiocytosis (FHL) is a rare hereditary disease that causes immune dysfunction and has an incidence of 1.2/1,000,000.^[1]^ It is induced by the mutations of various genes, including PRF1, UNC13D, and STX11 genes.^[2]^ Such mutations could lead to the cellular toxicity induced by natural killer cells and granules of T cells, the uncontrolled activity of immune cells, over-release of inflammatory cytokines, and finally, immune-mediated multiple organ injuries,^[3]^ among which injuries to the hematological system, liver, and spleen are the most prominent ones. Central nervous system (CNS) involvement can also occur^[4]^; however, it is very rare as the initial presentation. In this study, data of 2 patients with primary FHL induced by PRF1 gene mutation were analyzed. Both patients were from the same family and were with the initial presentation of CNS involvement. The reported findings could further the clinician’s understanding of this disease.

## 2. Case presentation

### 2.1. Case 1

The girl was first admitted to our hospital for an unsteady walk lasting for over 1 month at the age of 2 years and 8 months, and bilateral adduction of eyes for 7 days. The unsteady walk without obvious causes was observed 1 month before the hospitalization. Specifically, the girl could not walk straightly; she would fall easily down and could not go up- or down the stairs. The girl occasionally reported blurred vision, which lasted for several seconds and then was relieved, and bilateral adduction of eyes lasting for 7 days. The girl was born spontaneously at the term of G3P3, with a birth weight of 3.6 kg; her birth history and development were both normal. Her family history was as follows: both parents were healthy. The eldest brother was treated in our hospital for “headache and unsteady walk” 6 years ago and was diagnosed with “CNS infection, demyelinating disease, and metabolic acidosis.” By that time, the boy had already died. Physical examinations showed bilateral adduction of the eyes showed, while the abduction was limited. No obvious abnormalities were observed during rest.

**Figure 1.**
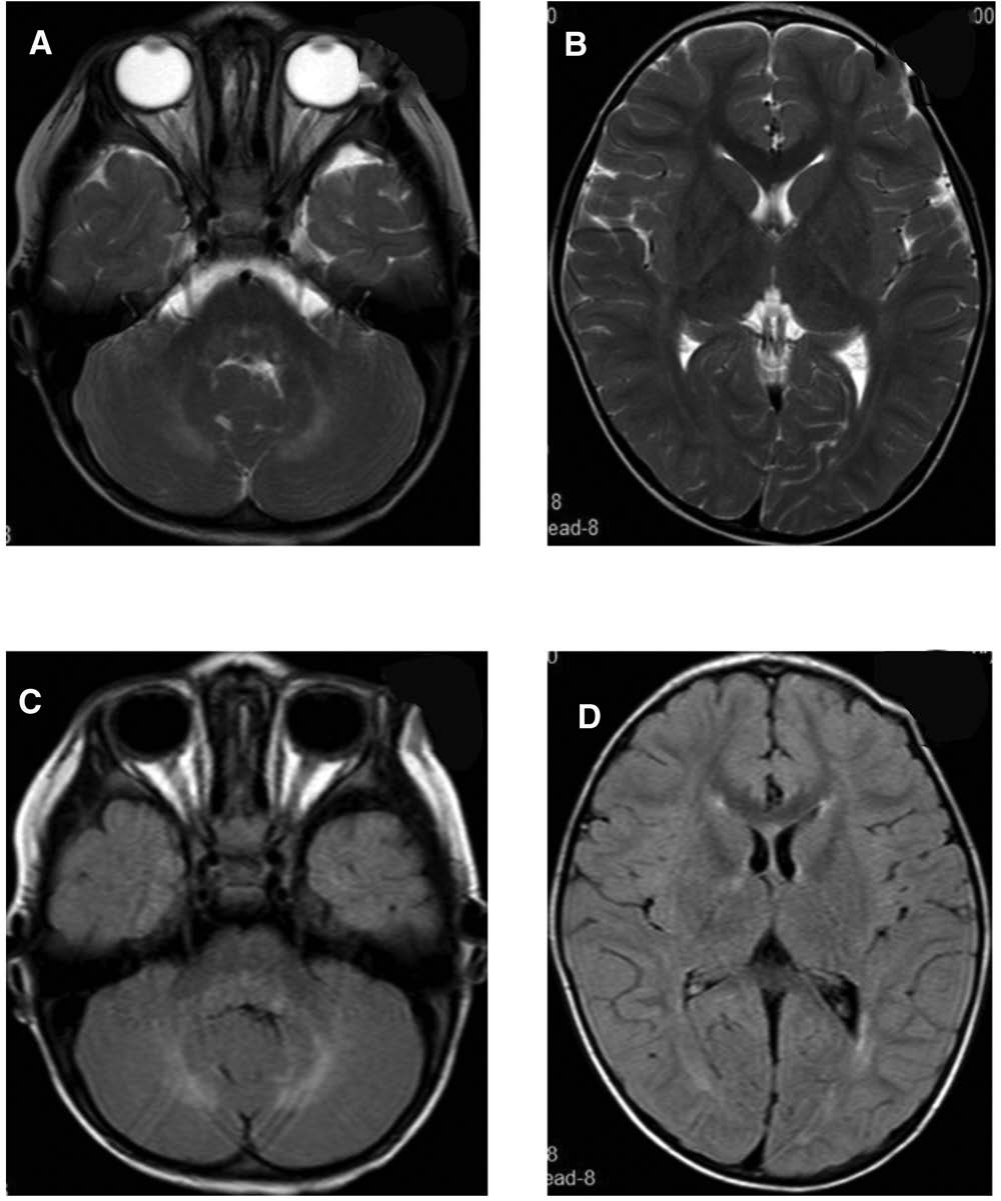
Cranial MRI of Case 1 at the age of 2 years and 8 months. Multiple patchy, slightly long T2 and FLAIR high signal shadows found at white matters of bilateral frontal, parietal, and occipital lobes, as well as basal ganglia, bilateral cerebellar hemispheres, and dorsal pons, with unclear boundaries. The posterior parts of the bodies of bilateral lateral ventricles were slightly widened. FLAIR = fluid-attenuated inversion recovery, MRI = magnetic resonance imaging.

Laboratory tests showed no abnormality. Cranial magnetic resonance imaging (MRI) showed multiple patchy, slightly long T2 and fluid-attenuated inversion recovery (FLAIR) high signal shadows at white matters of bilateral frontal, parietal, and occipital lobes, as well as basal ganglia, bilateral cerebellar hemispheres, and dorsal pons, which had unclear boundaries. The posterior parts of bodies of bilateral lateral ventricles were slightly widened (Fig. 1).

The girl was treated by levocarnitine (5 mL, twice/d), coenzyme Q10 (10 mg, 3 times/d), vitamin B1 tablet (10 mg, 3 times/d), vitamin B2 (5 mg, 3 times/d), and vitamin E (100 mg, once/d), and her symptoms gradually improved. Her walk became steady, bilateral adduction of eyes improved, and the eyes could be abducted to the midline. The girl was hospitalized and treated for 7 days, and once her condition improved, she was discharged.

**Figure 2.**
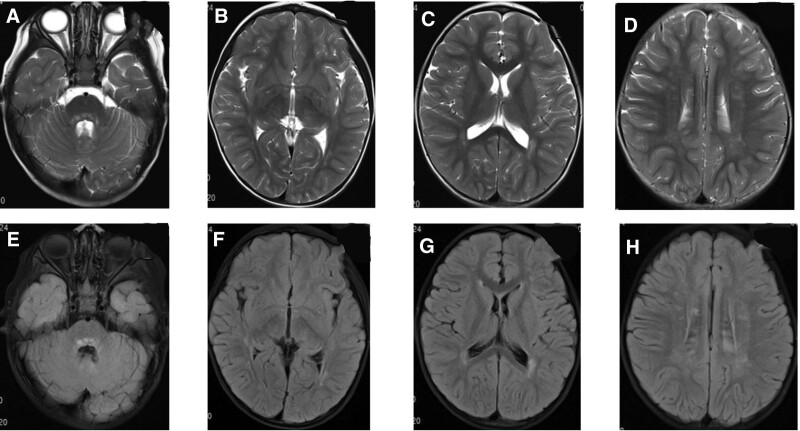
Cranial MRI of Case 1 at the age of 3 years. Multiple patchy and slightly long T1, long T2, and FLAIR high signal shadows found at white matters of bilateral frontal, parietal, and occipital lobes, as well as bilateral para-ventricular white matters, left thalamus, bilateral cerebellar hemispheres, and dorsal pons, with unclear boundaries. Some lesions showed small round shapes, some lesions were more limited than before, and some showed higher signals than before. The number of lesions in the left thalamus, dorsal pons, and bilateral lateral ventricles was higher than before. FLAIR = fluid-attenuated inversion recovery, MRI = magnetic resonance imaging.

When the girl was 3 years old, right eye adduction, in addition to diarrhea, appeared again. reexamination by cranial MRI (Fig. 2) showed multiple patchy and slightly long T1, long T2, and FLAIR high signal shadows at white matters of bilateral frontal, parietal, and occipital lobes, as well as bilateral para-ventricular white matters, left thalamus, bilateral cerebellar hemispheres, and dorsal pons, with unclear boundaries. Some lesions had small round shapes, some were more limited than before, and some showed higher signals than before. The number of lesions at left thalamus, dorsal pons, and bilateral lateral ventricles was higher than before. The posterior parts of bodies of bilateral lateral ventricles were slightly widened. The girl was given the same treatment as before, and her symptoms improved once more.

**Figure 3.**
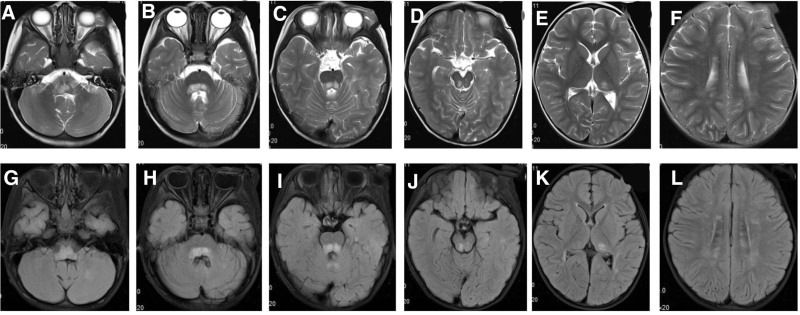
Cranial MRI of Case 1 at the age of 3 years and 2 months. Multiple patchy, slightly long T1, long T2, and FLAIR high signals found at white matters of bilateral frontal, parietal, and occipital lobes, as well as bilateral para-ventricular white matters, left thalamus, bilateral cerebellar hemispheres, and dorsal pons, with relatively clear boundaries are. Some lesions were small round shapes; some lesions of the left thalamus and dorsal pons had higher T2WI signals than before (March 24, 2021). The posterior parts of the bodies of bilateral lateral ventricles were slightly widened. FLAIR = fluid-attenuated inversion recovery, MRI = magnetic resonance imaging, T2WI = T2-weighted imaging.

**Figure 4.**
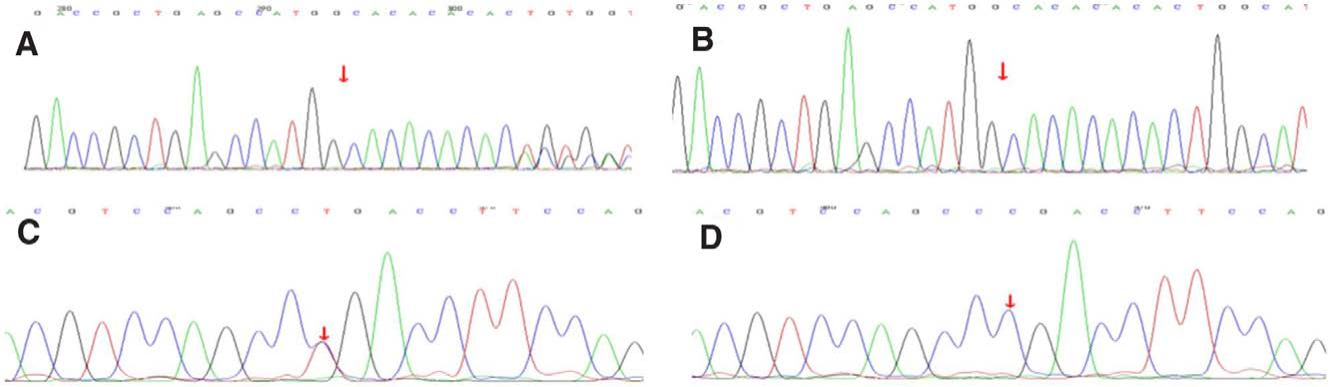
Genetic testing results of the Case 1. (A) Mutant genotype of c.1189_1190dupTG (p.H398Afs*23). (B) Mutant genotype of c.1189_1190dupTG (p.H398Afs*23). (C) Mutant genotype of c.394G>Ap.G132R. (D) Wild-type c.394G>Ap.G132R.

When the girl was 3 years and 2 months old, her left corner of the mouth became distorted; she had limited right abduction, as well as fever. The girl was re-admitted to the hospital for treatment. Cranial MRI (Fig. 3) showed multiple patchy, slightly long T1, long T2, and FLAIR high signals at white matters of bilateral frontal, parietal, and occipital lobes, as well as bilateral para- ventricular white matters, left thalamus, bilateral cerebellar hemispheres, and dorsal pons, whose boundaries were relatively clear. Some lesions had a small round shape, while lesions of the left thalamus and dorsal pons were wider than before (March 24, 2021), and T2-weighted imaging signals increased. The posterior parts of bodies of bilateral lateral ventricles were slightly widened. Genetic testing (Fig. 4) showed complex heterogeneous mutations of PRF1 gene, i.e. c.1189_1190dupTG (p.H398Afs*23) and c.394G>A (p.G132R). The girl was confirmed with type 2 familial FHL. According to the expert consensus on FHL issued in 2004, additional treatments by dexamethasone (10 mg/m^2^—6 mg, d 1–2 week), intrathecal injection of methotrexate + dexamethasone (once per week), and etoposide (150 mg/m^2^—90 mg, twice per week for 2 weeks) were also applied. After the treatment, the distortion of the left corner of the mouth and limited abduction of the right eye improved.

**Figure 5.**
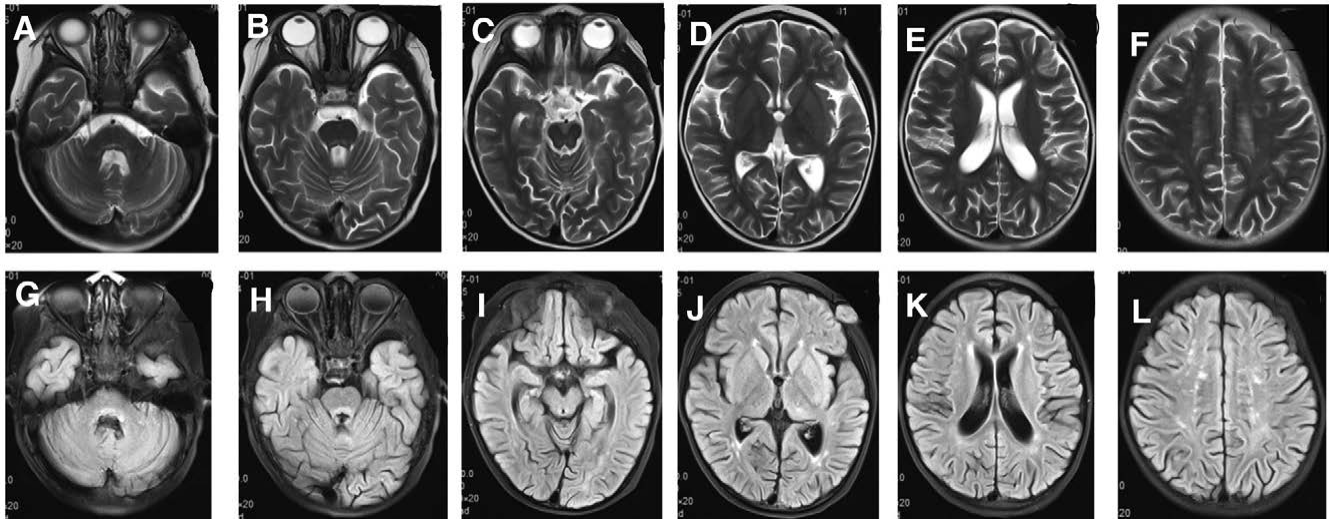
Cranial MRI of Case 1 at the age of 3 years and 4 months. Multiple patchy, slightly long T1, long T2, and FLAIR high signals found at white matters of bilateral frontal, parietal, and occipital lobes, as well as bilateral para-ventricular white matters, left thalamus, bilateral cerebellar hemispheres, and dorsal pons, with relatively clear boundaries. Some lesions had a small round shape. The lesions at bilateral cerebellar hemispheres, left thalamus, and dorsal pons was slightly smaller than before, and the FLAIR signals were reduced. However, the lesions of white matters of bilateral parietal and occipital lobes increased. The posterior parts of the bodies of bilateral lateral ventricles are slightly widened. FLAIR = fluid-attenuated inversion recovery, MRI = magnetic resonance imaging.

The girl underwent cranial MRI at the age of 3 years and 4 months (Fig. 5), which showed multiple patchy, slightly long T1, long T2, and FLAIR high signals at white matter of bilateral frontal, parietal, and occipital lobes, as well as bilateral para-ventricular white matters, left thalamus, bilateral cerebellar hemispheres, and dorsal pons, whose lesions were slightly smaller than before. FLAIR signals were also reduced than before. However, the lesions of white matters of bilateral parietal and occipital lobes were increased than before. The posterior parts of bodies of bilateral lateral ventricles were slightly widened.

**Figure 6.**
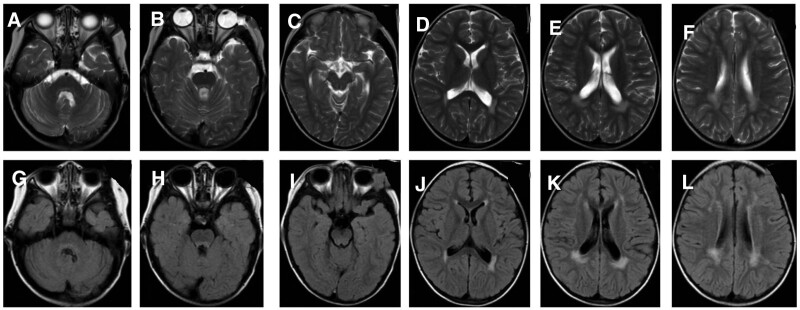
Some of the abnormal signals in the brain were slightly decreased than before.

Hematopoietic stem cell transplantation was performed at the age of 3 and 5 months. At present, the child is stable without further progression of the disease; however, she still has limited abduction of the right eye. The reexamination of head MRI at the age of 4 years (Fig. 6) showed multiple abnormal signals in the primary brain, some of which were slightly absorbed than before, but most of which showed slight change.

### 2.2. Case 2

Case 2 was the eldest brother of case 1. The boy was admitted to our hospital for intermittent headaches lasting for 5 days and unsteady walking for 1 day at the age of 4 years and 11 months. Intermittent headache without evident reasons, which was more prominent in the parietal lobe and occipital lobe, appeared 5 days before admission. The headache was not drastic and would spontaneously disappear. There was no fever, cough, wheezing, breathing suppression, vomit, or diarrhea. The boy had an unsteady walk and broad-based gait. He was capable of crouching, standing up, and walking. He had a respiratory infection history 10 days before the hospitalization. By the time of hospitalization, the cough and fever were already alleviated. The boy was born spontaneously at the term of G1P1, weighing 3.7 kg. His motor development after birth was normal. His family history was as follows: both parents were healthy and without consanguineous marriage. No genetic disease or infectious disease was reported in the family. Physical examinations showed bilateral adduction of the eyes, while the abduction was limited. Positive Romberg’s sign was found, and the boy did not cooperate in the finger-to-nose test. He showed a slightly drunken and broad-based gait.

**Figure 7.**
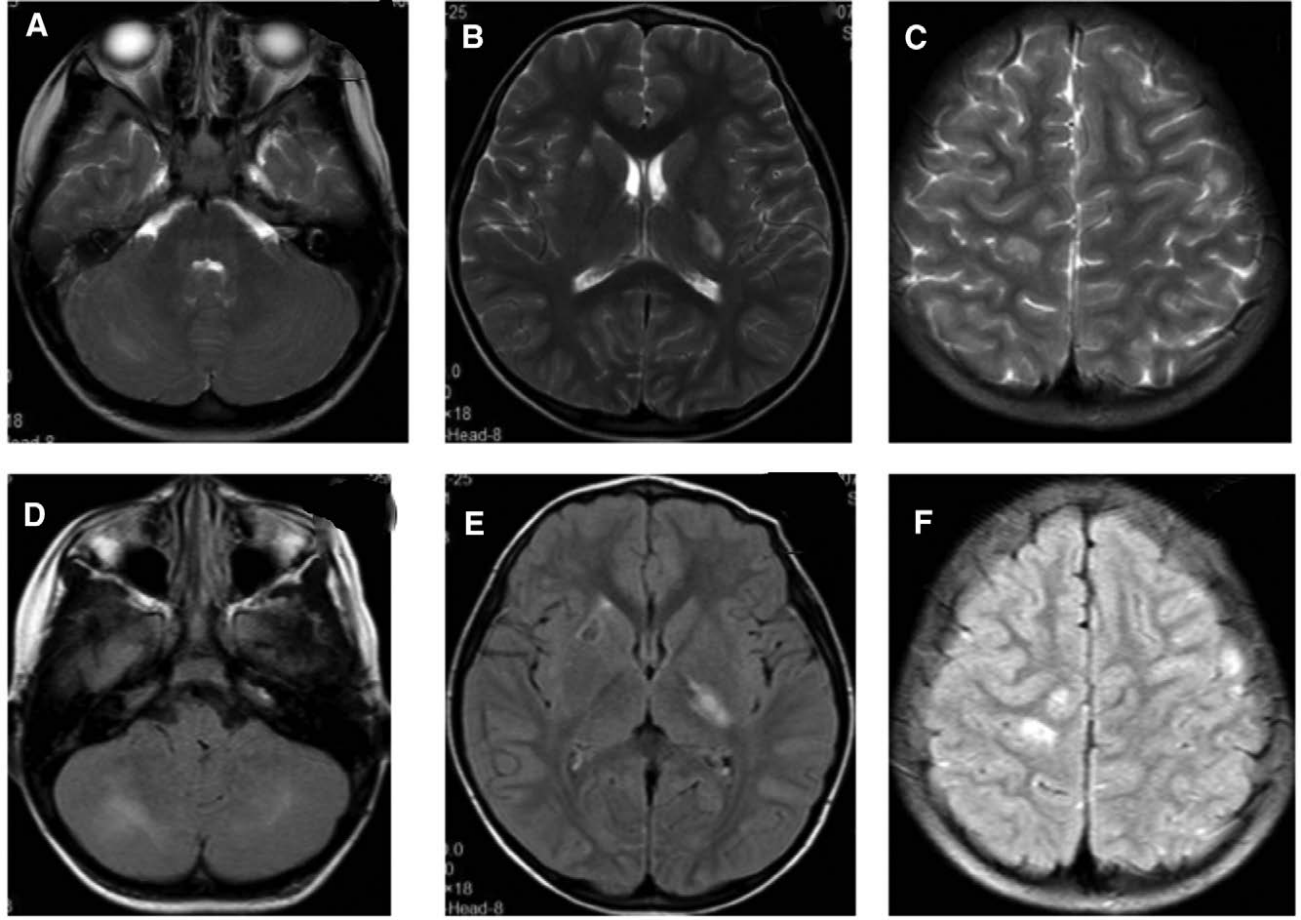
Cranial MRI of Case 2 at the age of 4 years and 11 months. Multiple abnormal signals found in bilateral cerebellar hemispheres, left basal ganglia, left temporal, and bilateral parietal regions. In addition, the encephalomalacia foci at the right basal ganglia could not be ruled out. MRI = magnetic resonance imaging.

CSF examination showed a white blood cell count of 111 × 10^9^/L, a mononuclear cell count of 108 × 10^9^/L, and a protein of 0.54 g/L. Oligoclonal IgG was positive in CSF. Immune encephalitis-related antibodies, paraneoplastic syndrome, and AQP4 were negative in blood and CSF. The titer of the mycoplasma pneumonia antibody in blood was 1:160. Video electroencephalogram showed persistent slow waves of high amplitudes with a continuous frequency of 2 to 3 Hz, which was significantly slower than in age-matched children. Cranial MRI showed multiple abnormal signals at bilateral cerebellar hemispheres, left basal ganglia region, left temporal lobe, and bilateral parietal lobe. The lesion of encephalomalacia at the right basal ganglia could not be ruled out (Fig. 7). The patient was diagnosed with: central nervous system infection and central nervous system demyelination disease. He was treated with Acyclovir, methylprednisolone sodium succinate, and immunoglobulin, after which the headache and unsteady walk improved. During the treatment, left distortion of the mouth corner was found when crying or laughing, the right eyelid could not be completely closed, the right nasolabial groove darkened, and the right forehead wrinkles also became dark, indicating right peripheral facial paralysis. Nerve nourishing and rehabilitation therapy were performed. The CSF was reexamined at 33 days of treatment, which showed that WBC was 19 × 10^9^/L, the mononuclear cell was 18 × 10^9^/L, and protein was 0.52 g/L. EEG showed normal results. The titer of the mycoplasma pneumonia antibody in blood was 1:160. The unsteady walk and right peripheral facial paralysis were alleviated, and the boy was discharged from the hospital.

**Figure 8.**
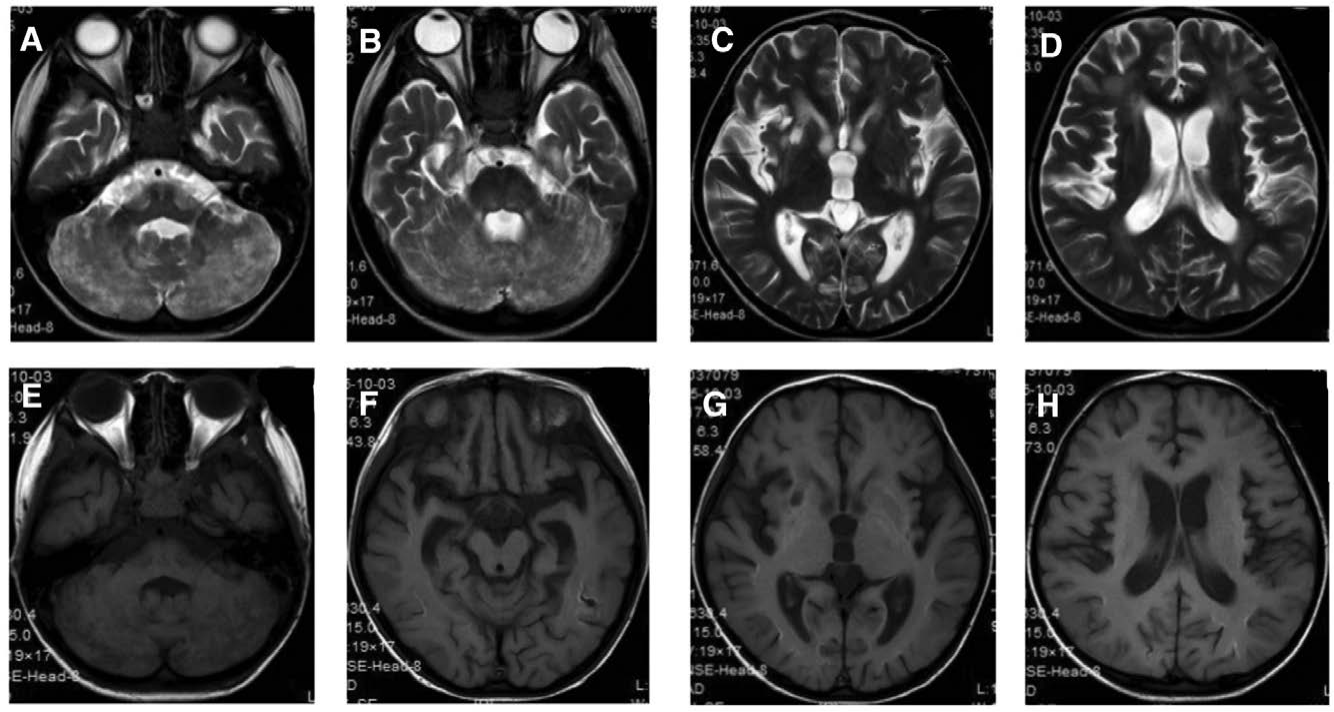
Cranial MRI of Case 2 at the age of 5 years and 9 months. Multiple sporadic intracranial abnormal signals accompanied by layer necrosis of the cortex. Also, the formation of partial encephalomalacia was seen. MRI = magnetic resonance imaging.

At the age of 5 years and 9 months, the boy experienced more than 10 convulsive seizures within 1 day, for which he was admitted to the hospital. After admission, the patient still had a fever and was unconscious. Cranial MRI showed multiple sporadic intracranial abnormal signals accompanied by layer necrosis of the cortex, as well as the formation of partial encephalomalacia (Fig. 8). The parents refused further treatment, and the boy was discharged from the hospital, after which he died.

**Figure 9.**
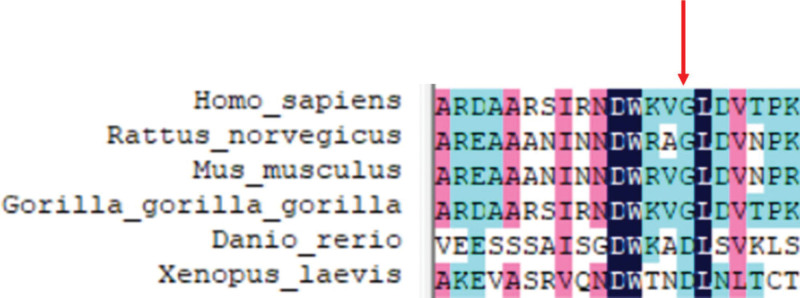
Conservative analysis of p.G132R showed that the G132 sequence is highly conservative in mammalians.

The testing showed 2 heterozygous mutations of the PRF1 gene, i.e., heterozygous mutation of c.1189_1190dupTG that induced frame-shifting mutation of an amino acid (p.H398Afs*23), and heterozygous mutation of G at 394 site to A (c.394G>A), leading to the variation of 132nd amino acid from glycine to arginine (p.G132R). Conservative analysis of p.G132R showed that the G132 sequence is highly conservative in mammalians (Fig. 9).

### 2.3. Pathogenesis analysis

c.1189_1190dupTG (exon3, NM_001083116) is a frame-shifting mutation leading to p.H398Afs*23 amino acid variation, which is a null mutation (frame-shifting mutation) that could lead to the loss of gene functions and is in agreement with PVS1. A database of previous studies already reported the cases of this site (Haemophagocytic lymphohistiocytosis, familial), which was labeled as DM (pathogenic mutation). The ClinVar database did not record the results of the pathogenicity of this site, which was in agreement with PM3_Strong. The frequency of this mutation in the database of a healthy population is 0.000004, which is a low-frequency mutation and is in agreement with PM2. The pedigree analysis demonstrated that the patients’ father carried the heterozygous mutation of this mutation, while the mother did not carry the mutation. According to the ACMG guidelines,^[5]^ the mutation was pathogenic.

**Figure 10.**
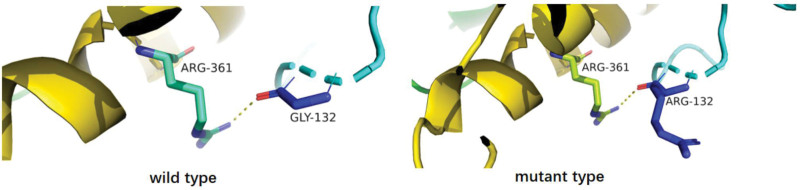
The amino acid at the 132nd site changed from GLY to ARG, which resulted in the mutation of nonpolar amino acids to a polar amino acid with a positive charge, leading to the polarity changes of the local protein. Mutation at this site could influence the protein structure and protein functions.

The c.394G>A (exon2, NM_001083116) mutation is a missense mutation that causes amino acid mutation of p.G132R. The amino acid at the 132nd site changes from GLY to ARG, which results in the mutation of nonpolar amino acids to a polar amino acid with a positive charge, eventually leading to the polarity changes in the local protein. The mutation at this site could influence the protein structure, consequently influencing the protein functions (Fig. 10). Previous studies already reported the cases with the recessive inheritance of this site (Haemophagocytic lymphohistiocytosis, familial), which was labeled as DM (pathogenic mutation). The ClinVar database did not record the results of the pathogenicity of this site, which was in agreement with PM3_Strong. This mutation is in the focal region, which is in agreement with PM1. The frequency of this mutation in the database of healthy populations is 0.0000083, which is a low-frequency mutation and is in agreement with PM2. According to the ACMG guidelines, the mutation was preliminarily considered a suspicious pathogenic mutation. In light of the clinical manifestations observed in children, the gene mutation was considered the pathogenic gene mutation.

## 3. Literature review

**Figure 11.**
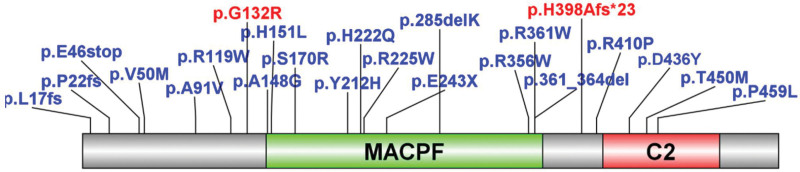
Sites of the involved genes in the domain. Blue fonts indicate mutations that were reported in other studies; red fonts indicate the mutations investigated in this study.

Wanfang, CNKI, and PubMed were searched for studies on children with hemophagocytic lymphohistiocytosis caused by PRF gene mutation using the search words “PRF1, Haemophagocytic lymphohistiocytosis, familial.” After reading the relevant literature, articles on children with central nervous system damage as the initial symptom were selected. Without the present 2 cases, 20 cases were previously reported. The sites of the involved genes in the domain are shown in Figure 11, and the clinical manifestations are shown in Table 1.

**Table 1:** Summary of clinical characteristics of PRF1 gene mutation-induced FHL cases with CNS injuries as the initial presentation.

Series no.	CNS manifestation									
	Ataxia	Convulsion	Encephalopathy	Limb paralysis	Eyeball movement disorder		Facial paralysis		Headache	
1^[6]^	+	−	+	−	−		−		+	
2^[6]^	−	+	+	−	−		−		+	
3^[7]^	−	+	+	+	−		−		−	
4^[8]^	+	+	+	+	+		−		−	
5^[8]^	+	+	+	+	−		−		−	
6^[9]^	+	+	+	+	+		−		−	
7^[9]^	+	+	+	+	−		−		−	
8^[10]^	+	−	−	+	+		−		+	
9^[11]^	−	−	−	+	+		−		+	
10^[12]^	−	−	−	+	+		−		+	
11^[12]^	+	+	+	+	−		−		−	
12^[13]^	+	+	−	−	+		−		+	
13^[14]^	−	+	−	+	−		−		−	
14^[15]^	−	+	−	−	+		−		+	
15^[16]^	−	+	+	+	−		−		−	
16^[17]^	+	+	+	+	−		+		−	
17^[17]^	+	+	+	−	−		+		+	
18^[17]^	+	+	+	+	−		−		+	
19^[17]^	+	+	+	−	−		−		−	
20^[18]^	−	−	−	+	−		m		−	
21	+	+	−	−	+		+		−	
22	+	+	−	−	+		+		−	
Total	14/22 (63.6%)	17/22 (77.3%)	13/22 (59.1%)	14/22 (40.9%)	9/22 (40.9%)		4/22 (18.2%)		9/22 (40.9%)	
Series no.	Cranial MRI presentation								Elevated WBC count in CSF	Genetic test results
	Cerebral hemisphere	Cerebellar hemispheres	Brainstem	Paraventricular white matter	Corpus callosum	Thalamus	Basalgangalia	Encephalatrophy		
1^[6]^	+	+	−	−	−	−	−	−	+	c.452A>T(p.H151L) c.666C>A(p.H222Q)
2^[6]^	+	+	−	−	−	−	−	−	NA	c.443C>G(p.A148G) c.666C>A(p.H222Q)
3^[7]^	+	−	−	+	−	−	−	−	−	c.50delT(p.17fs) c.1229G>C (p.R410P))
4^[8]^	+	+	+	+	+	−	−	−	+	c.1376C>T(p.P459L) c.1376C>T(p.P459L)
5^[8]^	+	+	−	+	−	−	−	−	+	c.1376C>T(p.P459L) c.1376C>T(p.P459L)
6^[9]^	+	+	−	+	−	−	−	−	+	c.673C>T (p.R225W) c.673C>T (p.R225W)
7^[9]^	+	+	+	+		+	+	+	+	c.673C>T (p.R225W) c.673C>T (p.R225W)
8^[10]^	+	+	+	−	−	−	−	−	−	c.673C>T (p.R225W) c.673C>T (p.R225W)
9^[11]^	NA	NA	NA	NA	NA	NA	NA	NA	NA	c.1066C>T(p.R356W) c.1349C>T(p.T450M)
10^[12]^	NA	NA	NA	NA	NA	NA	NA	NA	NA	c.394G>T(p.GLY132ARG) c.394G>T(p.GLY132ARG)
11^[12]^	+	+	−	−	−	−	−	−	+	c.148G>A(p.Val150MET) c.673C>T (p.R225W)
12^[13]^	+	+	−	−	−	−	−	−	−	(p.Glu46stop) (p. Ala91Val) (p.Arg119Trp)
13^[14]^	+	−	−	−	−	−	+	−	−	c.443C>C(p.A148G) c.666C>A(p.H222Q)
14^[15]^	+	+	+	−	+	−	−	−	+	c.1081A>T(p.ARG361TRP)
15^[16]^	+	+	+	−	−	−	−	−	+	c.508A>C(p.S170R) c.727G>T(p. E243X)
16^[17]^	+	+	+	−	−	−	+	−	+	c.634T>C(p.Y212H) c.1083_1094Del(p.361_364del)
17^[17]^	+	+	+	+	−	+	−	−	+	c.1349C>T(p.T450M) c.853_855del(p.285delK)
18^[17]^	+	+	−	−	−	−	−	−	+	c.1349C>T(p.T450M) c.1306G>T(p.D436Y)
19^[17]^	+	+	+	−	+	−	+	−	−	c.65delC(p.22Rfs*2) c.148G>A(p.V50M)
20^[18]^	+	−	+	−	−	−	−	−	+	c.673C>T (p.R225W) c.673C>T (p.R225W)
21	+	+	+	+	−	+	+	+	+	c.1189_1190dupTG(p.H398Afs*23),c.394G>A(p.G132R)
22	+	+	+	+	−	+	+	+	+	c.1189_1190dupTG(p.H398Afs*23),c.394G>A(p.G132R)
Total	20/20 (100%)	17/20 (85%)	11/20 (55%)	8/20 (40%)	3/20 (15%)	4/20 (20%)	6/20 (30%)	3/20 (15%)	14/19 (73.7%)	

− = no, + = yes, CNS = central nervous system, FHL = primary hemophagocytic phohistiocytosis, NA = data not available, MRI = magnetic resonance imaging.

## 4. Discussion and conclusions

Primary FHL is an autosomal recessive disorder that could be induced by the mutations of various genes such as PRF1, UNC13D, STX11, and STXBP2.^[19]^ The clinical manifestations include persistent fever, complete blood cell reduction, liver and spleen enlargement, and hemophagocytosis in bone marrow, liver, spleen, and lymph node tissues. The disease could also influence the CNS.

In their study, Blincoe et al^[20]^ investigated 38 hemophagocytic phohistiocytosis children with CNS injuries, whose median age of disease onset was 6.5 years and the median age of diagnosis was 28.3 months. The common manifestations included ataxia/gait abnormality (74%), epileptic seizure (53%), headache (47%), limb weakness or dyskinesia (42%), and visual abnormality (45%). Cranial MRI manifestations mainly included diffused multifocal white matter changes (79%), cerebellum white matter change (61%), paraventricular disorder, and brainstem disorder, edema, swelling, and volume reduction or atrophy. WBC count in CSF can be elevated (22/29), and proteins in CSF can be increased (15/29). Before the diagnosis was confirmed, CNS manifestations progressed or recurred in 74% of children. The disease in 14 patients progressed to systemic inflammatory disease (median time was 36.9 months after the occurrence of CNS infection). The treatment effect of stem cell transplantation was evident.

FHL2 induced by PRF1 gene mutation accounts for about 20-40% of all primary hemophagocytic phohistiocytosis.^[8,21]^ In this study, we reported on 2 children in the same family with primary hemophagocytic phohistiocytosis induced by PRF gene mutation. Both children were with the initial presentation of CNS injuries, and the initial presentation included an unsteady walk, eye movement disorder, and facial paralysis, mainly involving the cerebellum and brainstem. Convulsions gradually emerged in the children, and manifestations including disturbance of consciousness and encephalopathy occurred, indicating whole brain involvement. The involvement of cerebellum and brainstem involvement was relatively severe in initial imaging presentations and gradually progressed to subatentorial and supratentorial total brain injuries. The clinical manifestations were generally in agreement with the imaging progression.

Literature review showed that the clinical manifestations of children with hemophagocytic phohistiocytosis induced by PRF1 gene mutation mainly included cranial nerve injury (81.8%), convulsion (77.3%), ataxia (63.6%), encephalopathy (59.1%), and limb paralysis (40.9%). Cranial imaging mainly included the involvement of cerebral hemispheres (100%), cerebellar hemispheres (85%), brain stem (55%), and paraventricular white matter (40%), and 73.7% of children were with elevated WBC count in CSF. Of the confirmed patients, few were with inherent immune defects during the treatment,^[6]^ and genetic sequencing revealed PRF1 gene mutation. Doctors discovered some cases during academic communication, following which the diagnosis was confirmed by genetic testing.^[13]^ Most other cases underwent genetic testing during differential diagnosis to identify the mutant genes, after which the diagnosis was finally confirmed. Totally, 23 gene mutations have been discovered in 22 patients to date, including the 2 cases in this study. After excluding factors such as consanguineous marriage and the same pedigree, c.394G>A (p.G132R) occurred for 4 times, c.666C>A (p.H222Q) and c.1349C>T (p.T450M) occurred for 3 times each, c.1349C>T(p.T450M) occurred for 3 times, and c.443C>C(p.A148G) occurred for 2 times. These findings suggested that these mutations could be focal pathogenic mutations of this disease.

In this study, we reported 2 cases of primary hemophagocytic phohistiocytosis induced by PRF1 with the CNS injuries as initial manifestations, thus expanding the gene bank and phenotype spectrum. The literature review suggested that the involvement of the cerebellum and brain stem in patients with the initial presentation of ataxia and cranial nerve injury could be indicative of primary hemophagocytic phohistiocytosis. Innate immune examinations and genetic testing should be performed to early clarify the diagnosis, and stem cell transplantation should be performed to improve the prognosis.

## Author contributions

**Conceptualization:** Baoguang Li.

**Data curation:** Wenjuan Wu.

**Formal analysis:** Wenjuan Wu.

**Funding acquisition:** Baoguang Li.

**Investigation:** Wenjuan Wu.

**Methodology:** Baoguang Li.

**Project administration:** Baoguang Li.

**Resources:** Wenjuan Wu.

**Software:** Wenjuan Wu.

**Supervision:** Baoguang Li.

**Validation:** Baoguang Li.

**Visualization:** Yang You.

**Writing – original draft:** Yang You.

**Writing – review & editing:** Baoguang Li.
